# Triple nostrils in a calf

**DOI:** 10.1186/s13620-020-00173-z

**Published:** 2020-08-27

**Authors:** Takeshi Tsuka, Ai Saito, Yoshiharu Okamoto, Yuji Sunden, Takehito Morita, Ryo Nishimura, Yusuke Murahata, Kazuo Azuma, Masamichi Yamashita, Tomohiro Osaki, Norihiko Ito, Tomohiro Imagawa

**Affiliations:** 1 Clinical Veterinary Sciences, Joint Department of Veterinary Medicine, Faculty of Agriculture, Tottori University, 4-101, Koyama-Minami, Tottori, Japan; 2 Okayama Prefectural Federation Agricultural Mutual Aid Association, 1-30, Kuwata, Okayama-city, Okayama, Japan

**Keywords:** Calf, Computed tomography, Endoscopy, Magnetic resonance imaging, Triple nostrils

## Abstract

**Background:**

Nasal abnormalities are rare in bovines. In humans, nasal deformities are mainly classified as proboscis lateralis or supernumerary nostrils. This report discusses the etiology of triple nostrils in a calf, based on computed tomography, magnetic resonance imaging, and endoscopy.

**Case presentation:**

A female Holstein calf presented with triple nostrils. The following abnormalities were observed: (1) formation of a small and flat blind-ended middle nostril between the right and left nostrils; (2) presence of a hair-bearing surface on the muzzle; (3) abnormal curvature of the nasal septum, resulting in a narrower right nasal cavity due to transformation of the nasal bones; and (4) formation of a bone-like structure within the nasal septum. These findings were similar to those of supernumerary nostrils in humans.

**Conclusions:**

To the best of our knowledge, this represents the first description of a calf with triple nostrils. The use of imaging modalities is necessary for investigating the etiology of triple nostrils.

## Background

Two main types of congenital nasal malformations have been defined in newborn humans: proboscis lateralis, which is characterized by a soft-tissue tube- or trunk-like appendage projecting from the facial surface near the nose, and supernumerary nostrils, a condition characterized by the formation of accessory nostrils located near the two nostrils [1, 2]. Previous bovine cases have involved the formation of a tubular structure in the upper region of the normal nose, resembling proboscis lateralis in humans [1, 3]. Computed tomography (CT) and endoscopy were previously used for antemortem diagnoses of bovine nasal diseases [4, 5]. Magnetic resonance imaging (MRI) has been used for evaluating the skulls in bovines with brain diseases [6]; however, to our knowledge, MRI has not been used for antemortem evaluation of bovine nasal diseases. This report discusses the use of CT, MRI, and endoscopy in a bovine case characterized by a small-sized blind-end accessory nostril formed in the middle portion of the muzzle between the right and left nostrils. It also describes the surgical method used to remove the middle nostril, the strategy of which was developed based on the structural abnormality.

## Case presentation

**Fig. 1:**
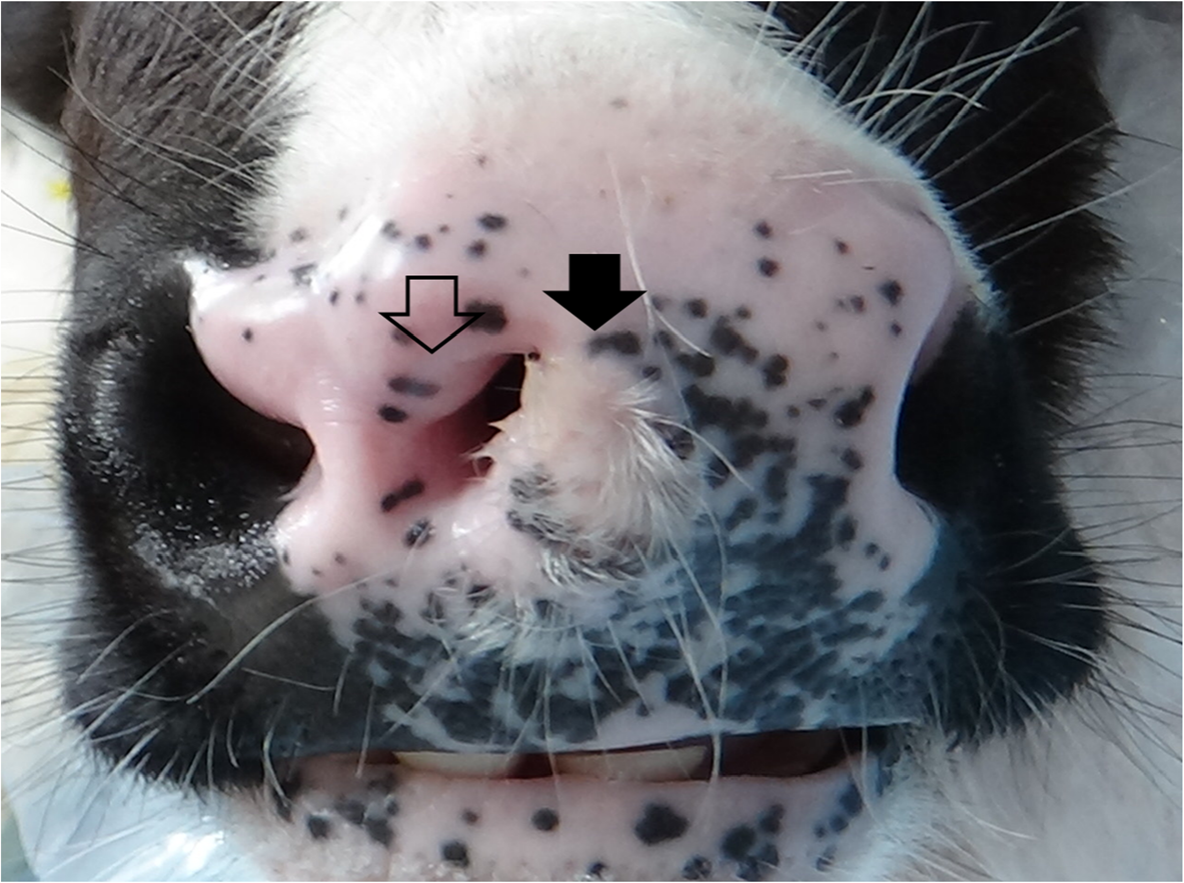
Macroscopic appearance of the nose of the 1-month-old female Holstein calf. A small, flat nostril (empty arrow) is evident within the region of the muzzle and between the right and left nostrils, which exit at the normal locations. A hair-bearing region (black arrow) is evident near the left edge of the middle nostril

**Fig. 2:**
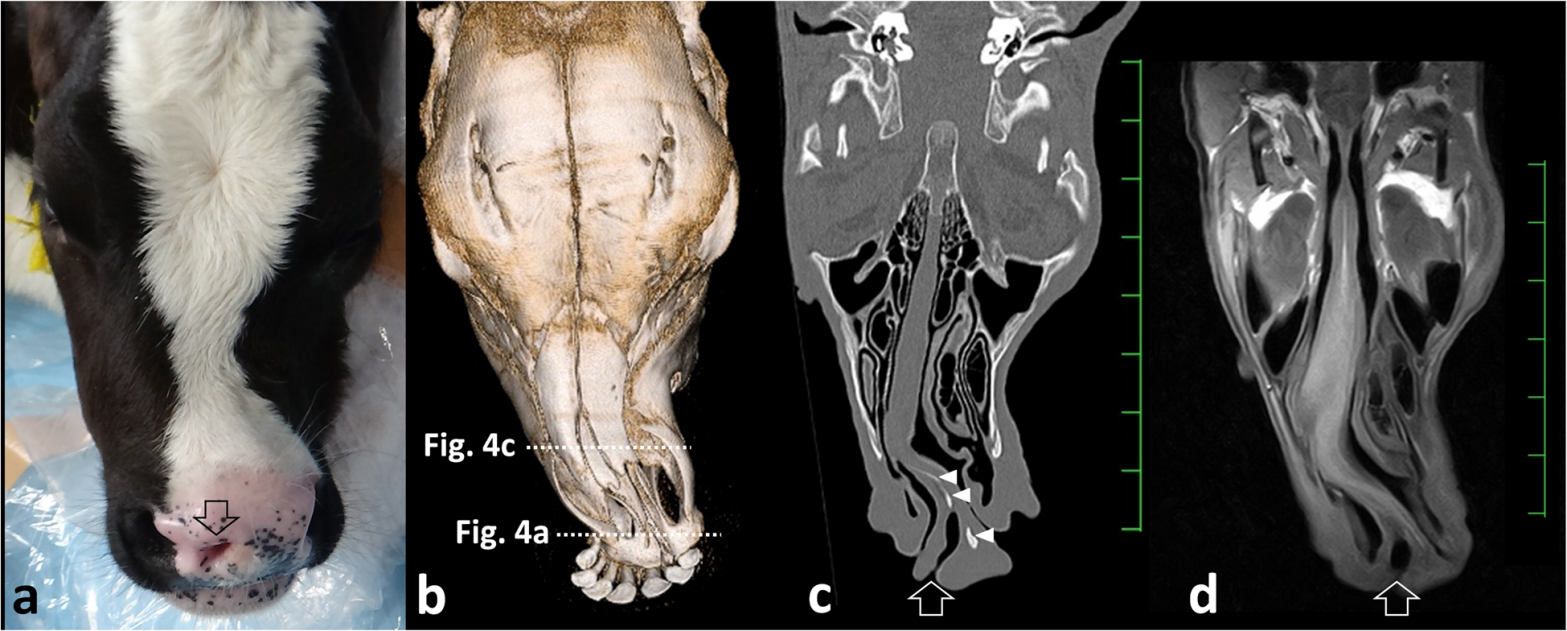
Dorsal macroscopic view of the nose (**a**), three-dimensional computed tomography (CT) visualizing the dorsal surface of the nasal bones (**b**), and dorsal CT (**c**) and T1-weighted MRI (**d**) visualizing the nasal cavity in the 1-month-old female Holstein calf. **a** The bridge of the nose is significantly curved toward the left. The middle nostril (empty arrow) is located in close proximity to the right nostril. **b** The transformation of the nasal bone allowed a curvature of approximately 30° from the central line of the nasal bone to the left. **c** The abnormal curve of the nasal septum allowed constriction within the right nasal cavity. The blind end is evident within the middle nostril (empty arrow). Bone-like structures (white arrowheads) are observed within the nasal septum. **d** A blind-ended middle nostril is evident (empty arrow), although a bone-like structure is not evident. Scale: 25 mm on CT and MRI

**Fig. 3:**
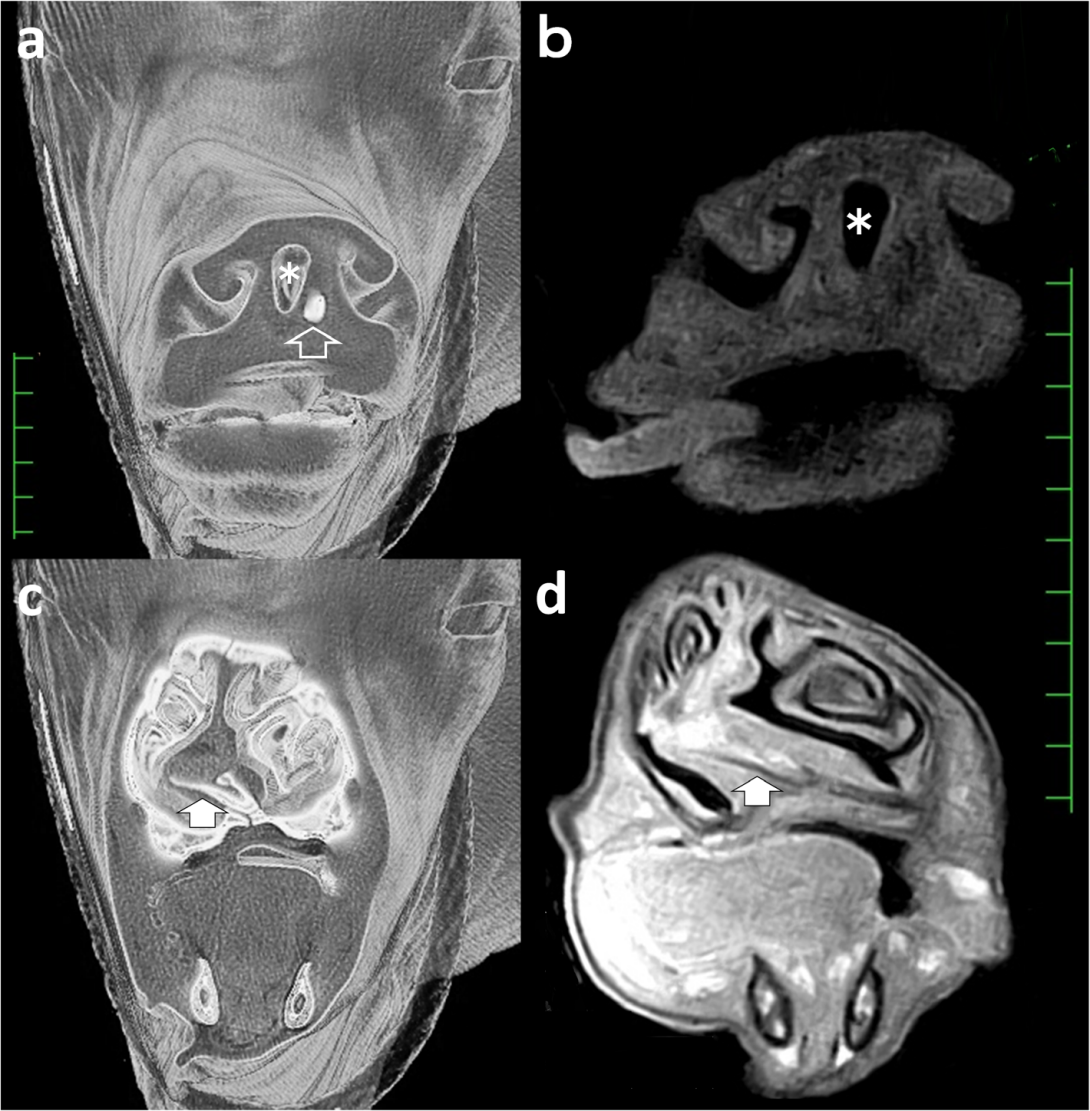
Transverse 3D-CT and T1-weighted MRI corresponding to approximately 1 cm (**a** and **b**) and 7 cm (**c** and **d**) deeper than the muzzle. **a** A bone-like structure (empty arrow) is observed in the left inferior site of the middle nostril (asterisk). **b** An elliptical middle nostril (asterisk) is seen in the space between the right and left nostrils. **c** The hyperdense vomer (white arrow) leans toward the right, along the transformed nasal septum. **d** The transformed vomer (white arrow) is imaged as a low-signal-intensity structure. The transformed turbinate bones are also clear. Scale: 10 mm on CT and MRI

**Fig. 4:**
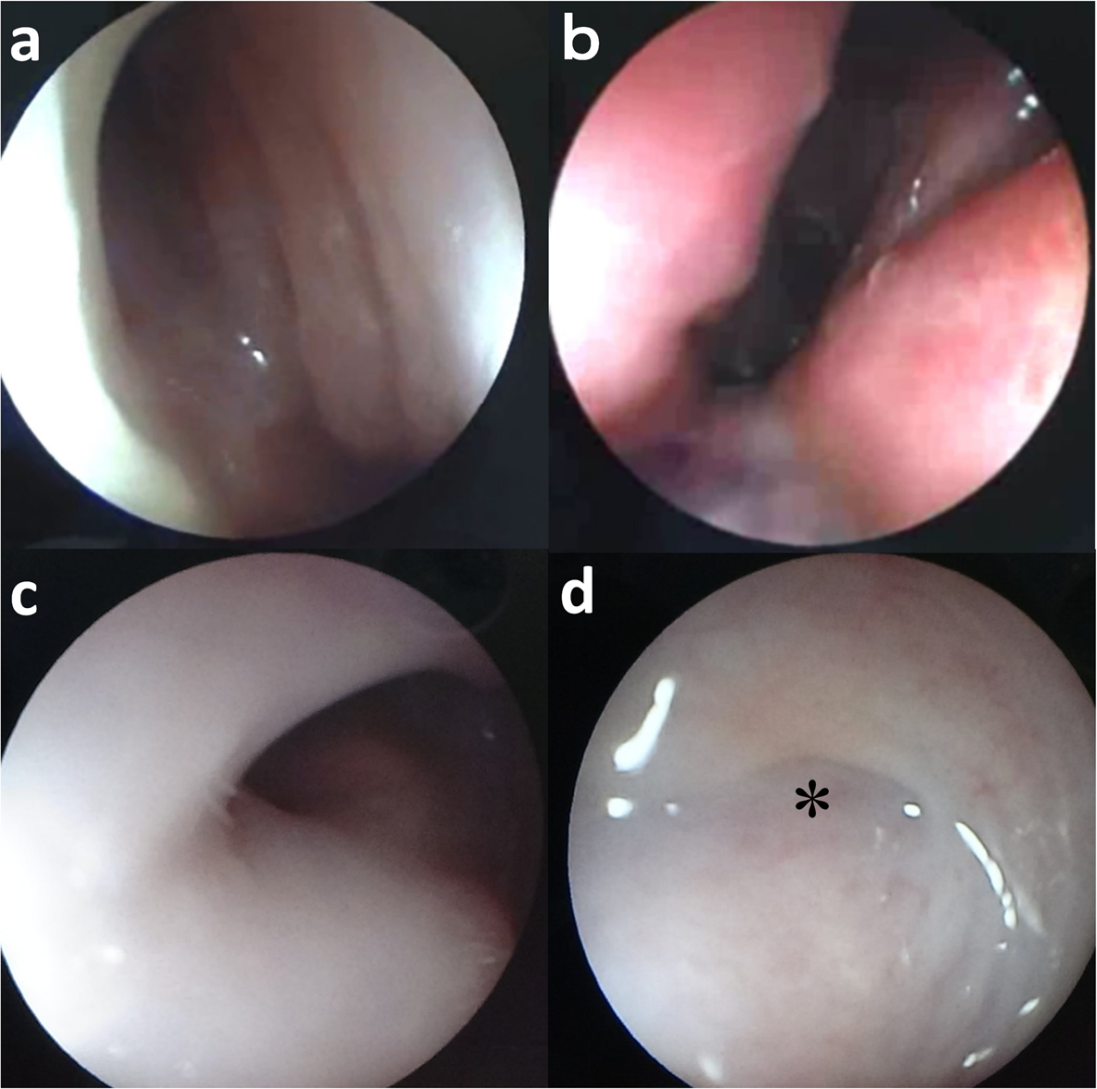
Endoscopy of the nasal cavities in the left nostril (**a**), the right nostril (**b**), the lumen near the opening (**c**), and the deepest site (**d**) of the middle nostril. **a** and **b** The lumen of the right nostril is narrower compared with that of the left nostril. **c** The mucosa is smooth and pale pink in the lumen of the middle nostril. **d** The blind-ended structure (asterisk) is observed approximately 5 cm deeper than the opening of the middle nostril

A female Holstein calf presented with a congenital formation of triple nostrils at birth. During the 1-month suckling period, the calf exhibited good weight gain and normal eating and drinking behavior. No respiratory signs, including cough or dyspnea, were evident. However, the calf was submitted to surgery for cosmetic improvement at the request of the owner. Of the three nostrils, the right and left existed in the normal locations within the nose (Fig. 1). These two nostrils were slightly smaller within a comparatively greater width of the muzzle than those in normal cattle. The middle nostril was located to the right of the center of the nose, in close proximity to the right nostril. This middle nostril was flat in the dorsoventral direction and smaller than the left and right nostrils. A hair-bearing region was found near the left edge of the middle nostril, a feature not normally seen on the muzzle. On the dorsal view, the bridge of the nose was running straight, with a sudden curve toward the left side (Fig. 2a). Under general anesthesia induced by intravenous injection of xylazine hydrochloride (0.1 mg/kg), and inhalation of 2–3% isoflurane via a tracheal tube, the calf underwent CT and MRI. A 16-section multidetector scanner (ECLOS; Hitachi, Tokyo, Japan) was used with X-ray tube settings of 120 kVp, a current of 175 mA, and scanning at a 0.625-mm-slice thickness for CT examination. For MRI examination, a low-field scanner (AIRIS Vento 0.3 T, Hitachi Medical Corporation, Tokyo, Japan) and a human knee coil were used. T1-weighted [time of repetition (TR), 450; time of echo (TE), 21; slice thickness, 5 mm] and T2-weighted images (TR, 3224; TE, 100; slice thickness, 5 mm) were obtained. Three-dimensional (3D) CT of the skull revealed that the left-sided nasal bone was more curved toward the left side at a third of the apex-caudal length of the nasal bone than the left side within the right-sided nasal bone (Fig. 2b). This transformation of the nasal bone allowed a curvature of approximately 30° from the central line of the nasal bone toward the left side. In addition, there was a minimal malocclusion because the transformed nasal and mandibular structures were slightly shorter than the maxillary bone within the apex. Transformation within the nasal bones may have been the skeletal cause of the malocclusion. Dorsal CT and T1-weighted MRI of the nasal cavity revealed that the nasal septum was abnormally curved along the transformed left-sided nasal bone, with a severe protrusion toward the right nasal cavity within the middle area of the nose (Figs. 2c,d). The abnormal curve of the nasal septum allowed constriction within the right nasal cavity, with a minimum lumen width of approximately 3 mm on CT. Of the three nostrils, the right and left nostrils were connected with the respective nasal cavities. The middle nostril ran obliquely from an opening within the muzzle toward the left side and backward within the deeper site. The lumen stopped in a blind-ended structure approximately 4 cm from the opening within the muzzle. On CT, a bone-like structure was seen running within the nasal septum from 5 mm deep through the muzzle to the bending point of the nasal septum. The blind-end of the middle nostril was located near this bone-like structure. Transverse 3D-CT and T1-weighted MRI revealed an elliptical middle nostril within the space between the right and left nostrils, approximately 1 cm deeper than the muzzle (Figs. 3a,b). A bone-like structure was observed in the left ventral side of the middle nostril on CT, however, MRI failed to disclose it. Transverse 3D-CT and T1-weighted MRI revealed that the nasal septum was thickened, markedly protruding toward the right nasal cavity at approximately 7 cm deeper than the muzzle (Figs. 3c,d). In addition, the vomer leaned toward the right, along the transformed nasal septum. The right-sided protruded bone of the vomer was longer than the left-sided protruded bone. The transformed conchae were evidently due to the transformed nasal septum. T1-weighted and T2-weighted MRIs revealed a normal brain appearance (data not shown). For internal observation of the lumina of the three nostrils, endoscopy [endoscope (EG-530NW, diameter 5.9 mm) and endoscopic machine (Advancia), FUJIFILM Medical Co., Ltd., Tokyo, Japan] was performed. Endoscopy of the left and right nostrils revealed that the right nostril had a narrower lumen than the left nostril (Figs. 4a,b). The constriction due to the curved nasal septum at 3 cm deep to the opening of the nostril made the passage of the endoscope difficult; however, the endoscope was finally passed through via the lumen. In the lumen of the middle nostril, the wall showed a normal mucosal structure with a pale pink and smooth surface (Fig. 4c). The blind-ended structure was observed approximately 5 cm deep toward the opening of the middle nostril (Fig. 4d). The wall of the blind-ended structure appeared to represent normal mucosa.

**Fig. 5:**
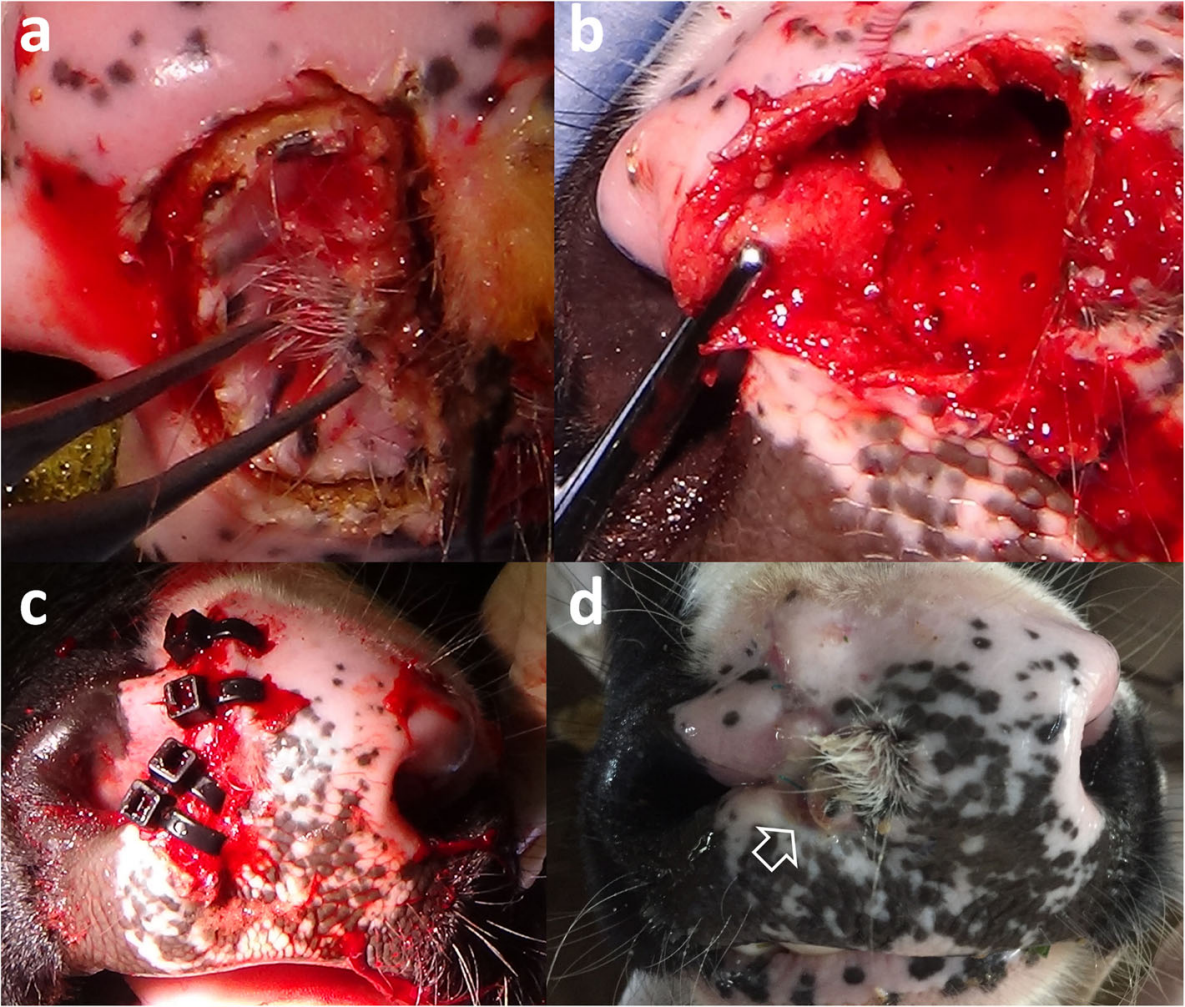
Intraoperative layout during removal of the middle nostril (**a** and **b**), and macroscopic layout of the nose soon after surgery (**c**) and at 1 month after surgery (**d**). **a** The muzzle is incised around the entire circumference of the edge of the middle nostril. **b** The middle nostril is removed (intact) by whole-circumference exfoliation toward the deepest part of the middle nostril. **c** The incision line on the muzzle is closed by clamping using four cable ties. **d** The incised line has healed, although irregularities are observed in a dorsoventral direction on the surface of the muzzle (empty arrow)

**Fig. 6:**
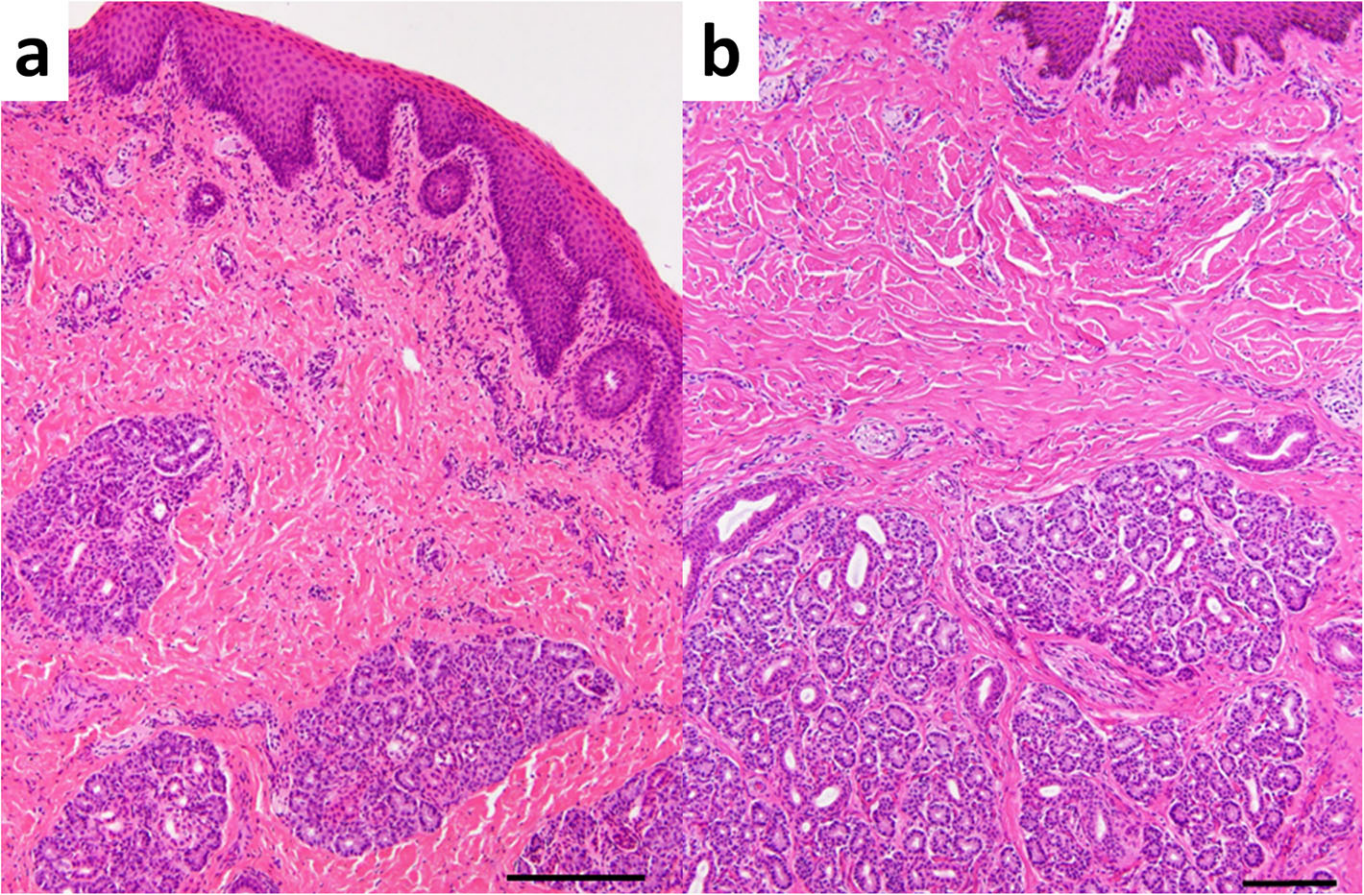
Histology of the middle nostril and hair-bearing surface of the muzzle in the present case (**a**) and the mucosal surface of the muzzle and nostrils in a normal, age-matched calf (**b**). **a** The tissue comprises normal skin structures, including nasal glands and subcutaneous connective tissues (H&E, bar = 250 μm). **b** Compared with the present case, the glandular lobes are larger in the middle nostril of the age-matched calf (H&E; bar = 100 μm)

Surgery was performed soon after CT, MRI, and endoscopic examinations. The muzzle was incised around the entire circumference and along the edge of the middle nostril using an electric scalpel (Fig. 5a). The incised region included part of the hair-bearing region. The submucosal tissue of the middle nostril was dissected bluntly, or by the electric scalpel as appropriate, toward the deep part of the incised region. Whole-circumference exfoliation toward the deepest part of the middle nostril allowed intact removal of the middle nostril (Fig. 5b). After surgical removal, the defect was sutured from the deepest part toward the shallower part with a monofilament absorbable suture; the suture joined the left and right lateral walls of the defect. After closure of the dead space under the surface of the muzzle, four cable ties were passed through small incisions made in both sides near the incision line of the muzzle and clamped to close the incision line (Fig. 5c). After surgery, systemic antibiotics were administered for 7 days. No postoperative complications were observed, and the cable ties were removed after 14 days. The incised line healed; however, irregularities were observed along the dorsoventral direction as a trace of the incised line on the surface of the muzzle (Fig. 5d). Within the central region of the muzzle, hair growth was observed again. This animal did not exhibit any respiratory abnormality, despite kept the narrow lumen of the right nasal cavity and the transformed bridge of the nose. The excised middle nostril was fixed in 10% neutral-buffered formalin solution and embedded in paraffin wax. Sections were cut at a thickness of 5 μm and stained with hematoxylin and eosin. Histologically, the surgically removed tissue consisted of normal nostril structures, including nasal glands, skin adnexa, and a few sinus hairs (Fig. 6). However, the glandular lobes were smaller than those of a control nostril from an age-matched calf (which had died because of digestive disease); however, there was no difference between the present case and the age-matched calf with regard to tissue components and structure.

## Discussion and conclusions

The present case involved a female Holstein calf with a small, blind-ended nostril located between two normal nostrils, near the middle of the nose. In bovines, congenital malformations of the nostrils are extremely rare, and they have previously only been described as a tubular structure in the upper region of the normal nose, with a nostril-like hole on the top of the tubular structure [3]. The previously reported lesion may be the bovine equivalent of proboscis lateralis, which occurs in 1:1,000,000 to 1:100,000 of human newborns and is characterized by a soft-tissue tube- or trunk-like appendage projecting from the surface of the face, near the nose [1]. On the other hand, the present findings may be analogous to supernumerary nostrils, which may result from an abnormal formation of the nose and nasal cavity during embryogenesis due to duplication of the nasal placodes (two ectodermal thickenings arising on each side of the dependent part of the frontonasal process) or fissuring of the lateral nasal process (in which the alae of the nose are formed together with the primitive nasal cavity) [7]. The abnormality of the nose observed in the present case was characterized by the following features: (1) presence of a hair-bearing region, histologically resembling normal hair-bearing skin located on the muzzle between the middle and left nostrils; (2) formation of a bone-like structure emanating from the deep parts of the hair-bearing region of the muzzle to the nasal septum and separating the middle and left nostrils; (3) abnormal curvature of the nasal septum, resulting in a narrower right nasal cavity; and (4) deformation of the nasal bone. These features indicate the following anomalous processes: (1) premature development, with the formation of a pair of nostrils (resulting in the right and middle nostrils) within the right nasal bud during the development of the nasal placode (bud); and (2) fusion of the right and left nasal buds, resulting in the left nostril. Premature development of the right nasal placode may have induced the formation of the bony tissue and skin for the formation of the normal nasal structures before embryological fusion. Such lesions have been observed in a small proportion of human cases, and left-sided unilateral or bilateral involvement of accessory nostrils is typical in supernumerary nostrils [2].

In humans, supernumerary nostrils are classified into three types, based on accessory nostril shape and location: type 1, a smaller accessory nostril located exterior to a pair of normal-sized nostrils; type 2, a smaller accessory nostril sandwiched between a pair of normal-sized nostrils and located near the midline of the nose; and type 3, two smaller nostrils located unilaterally and a normal-sized nostril on the opposite side of the nose [8]. The present bovine lesion is more similar to a human type-2 nostril malformation [8]. Intact resection of the accessory nostril is the recommended treatment for type-2 lesions [8].

In the present case, the middle nostril was a blind-ended structure. Openings into the nasal cavity are frequently found within abnormal nostrils in human cases of supernumerary nostrils, and a minority of the cases exhibit blind-ended structures [9]. Blind-ended lesions are less likely to result in respiratory symptoms but may become subject to infection, because the muzzle is widely exposed to a range of microorganisms in the environment. Surgical removal of an abnormal nostril may therefore be required in bovine cases with blind-ended lesions.

CT was superior to MRI for diagnosis of this disease, because the morphological abnormality of the nose was well characterized using CT-based 3D reconstruction. The 3D reconstruction has been utilized for diagnosis of non-syndromic congenital cleft lip and jaw; the unilateral type of this disease involved a nasal curvature, resembling that of the present case [5]. In addition, contrary to MRI, CT disclosed the bone-like structure formed within the nasal spectrum and facilitated the evaluation of the etiology of this disease. Endoscopy also enables a real-time view, as the endoscope is advanced into areas of the cavity, and in general enables good visualization of the nasal cavity. Endoscopy can also be utilized for biopsy guidance. MRI may facilitate the visualization of intranasal structures (such as the turbinate bones) and parenchymal lesions by distinguishing these from nasal discharge and may help evaluate any invasion of the intracranial cavity by nasal lesions via the destroyed cribriform plate [6]. The use of imaging modalities is critical to identifying the openings of abnormal nostrils into the nasal cavity when evaluating the need for surgical treatment.

## Supplementary information

**Additional file 1.** Computed radiography images (REGIUS Console CS-3, Konica Minolta Health Care, Japan). (a) Ventrodorsal radiograph of the maxilla reveals a radiolucent airway in the tubular lumen of the middle nostril (empty arrow). The structure of the lumen is unclear in the deeper area of the middle nostril because it is superimposed by the radiopaque bone body of the mandible. The airway in the lumen of the left nostril leans toward the right along the curved nasal septum (empty arrowheads). (b) On the ventrodorsal intraoral radiograph of the maxilla, taken while a radiographic cassette is inserted into the opened mouth, a blind-ended radiolucent structure is evident in the lumen of the middle nostril (empty arrow). The nasal septum is curved toward the right (empty arrowheads), resulting in a narrow radiolucent lumen of the right nostril. Scale = 10 mm.

**Additional file 2. **The dorsal T1-weighted magnetic resonance images (MRIs) provide a more dorsal view than the section shown in Fig. 2 (a and b). The sagittal T1-weighted and T2-weighted MRIs of the brain show the levels of the left olfactory bulb (c and d) and the right olfactory bulb (e and f). The transverse T1-weighted and T2-weighted MRIs of the brain show the lateral ventricle at the superior portion of the hippocampus and the third ventricle at the inferior portion of the hippocampus, separated into two parts by the interthalamic bridge (g and h). (a) The nasal septum (NS) is not abnormally curved, and it runs along the midline in the caudal level of the nasal cavity. (b) The structures of the left and right olfactory bulbs (OB) are symmetric. (c–f) The structures of the left and right olfactory bulbs (OB) are normal. Moreover, abnormality is not evident in the midline of the cerebrum, brainstem, or cerebellum. (g and h) The symmetry in the cerebral hemisphere is not abnormal in shape on the T1-weighted image or in the signal intensity of the gray and white matters on the T2-weighted image. The ratio of the size of the cerebral ventricle (V) (including the lateral and third ventricles) to the size of the cerebrum (C) is 0.096 (9.6%). The VC ratio is close to the value in healthy Holstein calves (0.082 ± 0.039; *n* = 26) and lower than 0.15, which is indicative of severe ventricular dilation associated with the neurological signs (*n* = 11) [6]. Scale = 10 mm on MRIs.

## Data Availability

Not applicable.

## Abbreviations

CT: computed tomography
MRI: magnetic resonance imaging
TE: time of echo
TR: time of repetition
3D: three-dimensional
