# Supplementary figures and images for: Triple nostrils in a calf
Source: Ir Vet J. 2020 Aug 27;73:19. doi: 10.1186/s13620-020-00173-z (PMC7453521; doi:10.1186/s13620-020-00173-z)

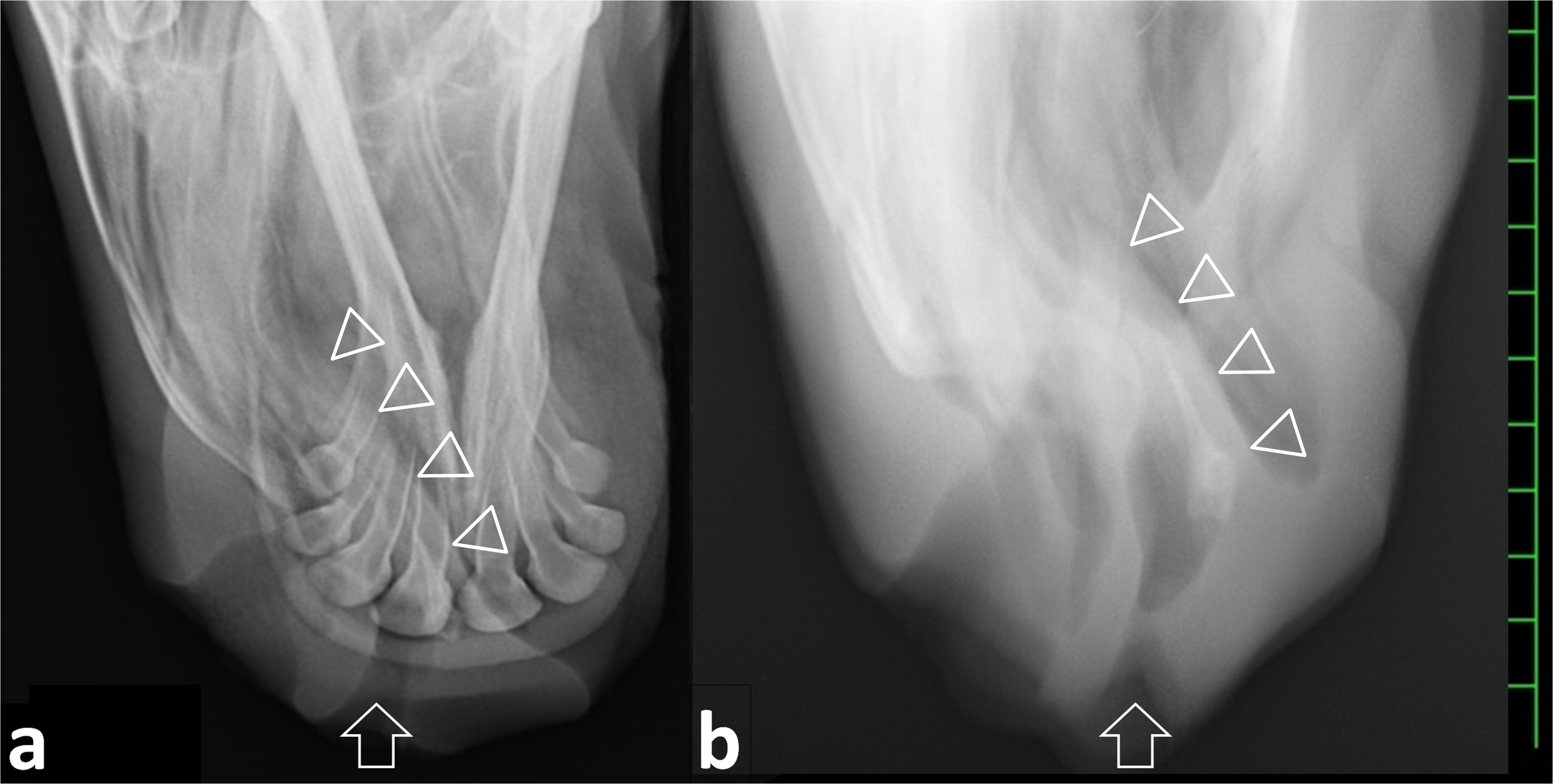

Supplement: Supplementary file 1 — Additional file 1. Computed radiography images (REGIUS Console CS-3, Konica Minolta Health Care, Japan). (a) Ventrodorsal radiograph of the maxilla reveals a radiolucent airway in the tubular lumen of the middle nostril (empty arrow). The structure of the lumen is unclear in the deeper area of the middle nostril because it is superimposed by the radiopaque bone body of the mandible. The airway in the lumen of the left nostril leans toward the right along the curved nasal septum (empty arrowheads). (b) On the ventrodorsal intraoral radiograph of the maxilla, taken while a radiographic cassette is inserted into the opened mouth, a blind-ended radiolucent structure is evident in the lumen of the middle nostril (empty arrow). The nasal septum is curved toward the right (empty arrowheads), resulting in a narrow radiolucent lumen of the right nostril. Scale = 10 mm. [file 13620_2020_173_MOESM1_ESM.tif]

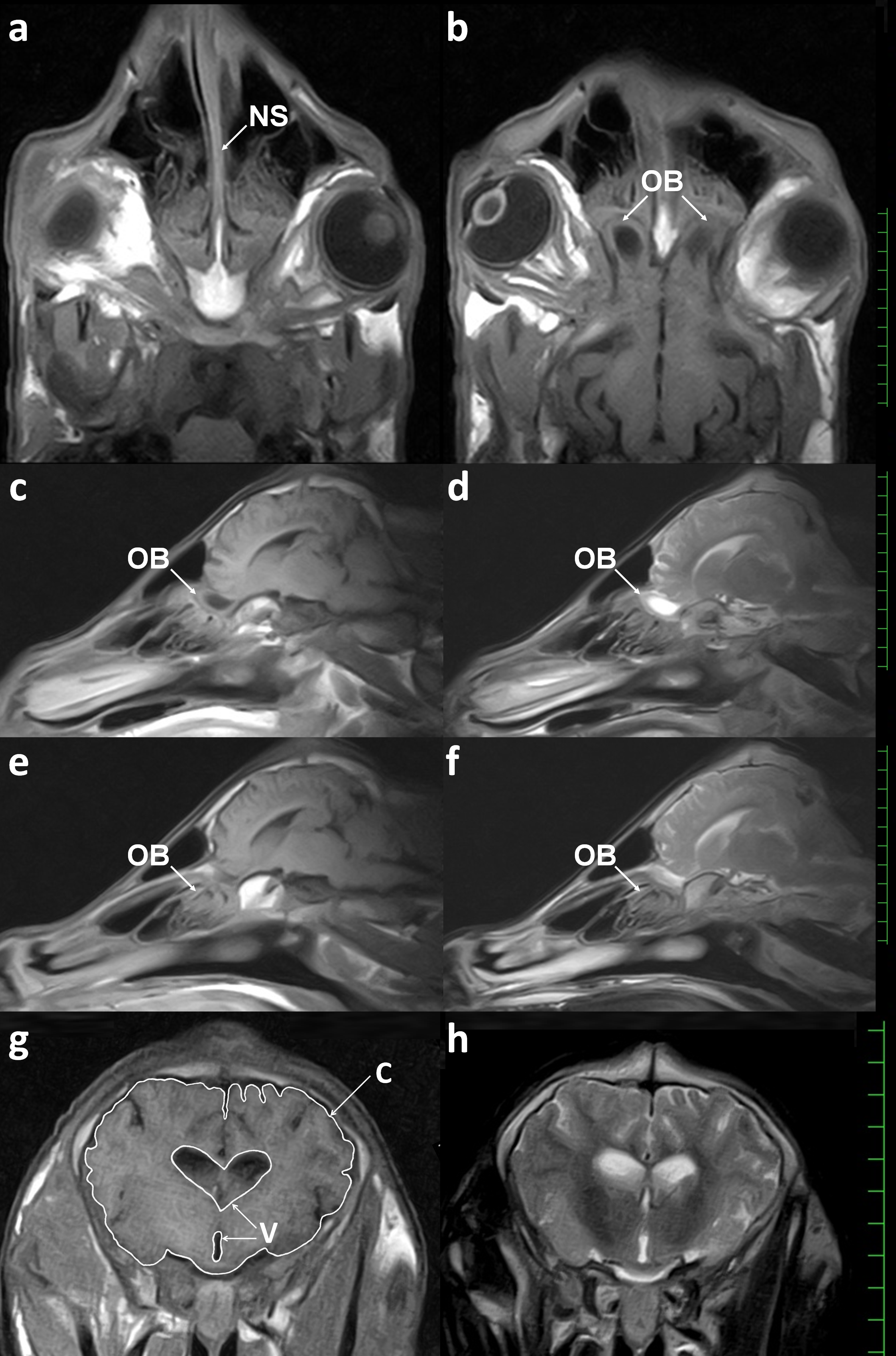

Supplement: Supplementary file 2 — Additional file 2. The dorsal T1-weighted magnetic resonance images (MRIs) provide a more dorsal view than the section shown in Fig. 2 (a and b). The sagittal T1-weighted and T2-weighted MRIs of the brain show the levels of the left olfactory bulb (c and d) and the right olfactory bulb (e and f). The transverse T1-weighted and T2-weighted MRIs of the brain show the lateral ventricle at the superior portion of the hippocampus and the third ventricle at the inferior portion of the hippocampus, separated into two parts by the interthalamic bridge (g and h). (a) The nasal septum (NS) is not abnormally curved, and it runs along the midline in the caudal level of the nasal cavity. (b) The structures of the left and right olfactory bulbs (OB) are symmetric. (c–f) The structures of the left and right olfactory bulbs (OB) are normal. Moreover, abnormality is not evident in the midline of the cerebrum, brainstem, or cerebellum. (g and h) The symmetry in the cerebral hemisphere is not abnormal in shape on the T1-weighted image or in the signal intensity of the gray and white matters on the T2-weighted image. The ratio of the size of the cerebral ventricle (V) (including the lateral and third ventricles) to the size of the cerebrum (C) is 0.096 (9.6%). The VC ratio is close to the value in healthy Holstein calves (0.082 ± 0.039; n = 26) and lower than 0.15, which is indicative of severe ventricular dilation associated with the neurological signs (n = 11) [6]. Scale = 10 mm on MRIs. [file 13620_2020_173_MOESM2_ESM.tif]
